# Exploring individual and demographic characteristics and their relation to CHNRI Criteria from an international public stakeholder group: an analysis using random intercept and logistic regression modelling

**DOI:** 10.7189/jogh.09.010701

**Published:** 2019-06

**Authors:** Kerri Wazny, Niall Anderson, Diego G Bassani, John Ravenscroft, Kit Yee Chan, Igor Rudan

**Affiliations:** 1 Centre for Global Health Research, Usher Institute of Informatics and Population Health Sciences, University of Edinburgh, Edinburgh, UK; 2 Centre of Population Health Sciences, Usher Institute of Informatics and Population Health Sciences, University of Edinburgh, Edinburgh, UK; 3 Department of Pediatrics, Hospital for Sick Children and University of Toronto, Toronto, ON, Canada; 4 Centre for Global Child Health and SickKids Research Institute, Hospital for Sick Children, Toronto, ON, Canada; 5 Moray House School of Education, University of Edinburgh, Edinburgh, UK

## Abstract

**Introduction:**

The Child Health and Nutrition Research Initiative (CHNRI) method for health research prioritisation relies on stakeholders weighting criteria used to assess research options. These weights in turn impact on the final scores and ranks assigned to research options. Three quarters of CHNRI studies published to date have not involved stakeholders in criteria weighting. Of those that have, few incorporated members of the public into stakeholder groups. Those that have compared different stakeholder groups, such as donors, researchers, or policy makers, showed that different groups place different values upon CHNRI criteria. When choosing the composition of a stakeholder group, it may be important to understand factors that may influence weighting. Drawing upon a group of international public stakeholders, this study explores some of the effects of individual and demographic characteristics has on the weights assigned to the most commonly used CHNRI criteria, with the aim of informing future researchers on avoiding future biases.

**Methods:**

Individual and demographic information and 5-point Likert scale responses to questions about the importance of 15 CHNRI criteria were collected from 1031 “Turkers” (Amazon Mechanical Turk workers) via Amazon Mechanical Turk (AMT), which is an online crowdsourcing platform. Thirteen of the fifteen criteria were analysed using random-intercept models and the remaining two were analysed through logistic regression.

**Results:**

Self-reported health status explained most of the variability in participants’ responses across criteria (11/15 criteria), followed by being female (10/15), ethnicity (9/15), employment (8/15), and religion (7/15). Differences across criteria indicate that when choosing stakeholder groups, researchers need to consider these factors to minimise bias.

**Conclusion:**

Researchers should collect and report more detailed information from stakeholders, including individual and demographic characteristics, and ensure participation from both genders, multiple ethnicities, religious beliefs,

and people with differing health statuses to be transparent regarding possible biases in health research prioritisation. Our analyses indicate that these factors do influence the relative importance of these values, even when the data appears fairly homogeneous.

The Child Health and Nutrition Research Initiative (CHNRI) research prioritisation method involves having researchers generate and evaluate research options (ie, research questions or ideas) against pre-established criteria [1]. These criteria are, in turn, evaluated by a wider stakeholder group, which sets weights for each criterion, and these are used to calculate the final relative ranking for the research priorities. Wider stakeholder involvement is a key part of the CHNRI process; however, over three quarters of published CHNRI exercises have not used a wider stakeholder group to weight criteria and instead presented crude scores [2]. This was posited to be due to those leading the studies preferring to present crude values rather than use weighted values from an unrepresentative group of stakeholders [2].

Stakeholder involvement in research prioritisation has been described as an indispensable part of the research prioritisation process [3]. If the intention of including opinions of wider stakeholders is to reflect their values, it is important to ensure the values of stakeholder groups of varying demographics are reflected. Indeed, previous Child Health and Nutrition Research Initiative (CHNRI) research prioritisation exercises have found differences in research prioritisation from researchers depending on their geographic locations [4,5], and on which CHNRI criteria different stakeholder groups value most [6].

When choosing who would make up a stakeholder group, it may be important to understand what characteristics should be taken into account to promote representativeness, and which ones may influence weights. Understanding this will enable future researchers conducting CHNRI exercises to know which characteristics are important to obtain a balance of when developing a stakeholder group. As part of a larger exercise to use the public to set stakeholder values from the public, our objective is to explore the association between individual and demographic characteristics of a crowd of laypersons and the most commonly used CHNRI criteria, using Amazon Mechanical Turk (AMT), a web-based crowdsourcing platform, for collecting responses.

## METHODS

This study is nested in another, which sought to re-weight criteria for the Child Health and Nutrition Research Initiative’s (CHNRI) research priority setting method [7].

### Data collection

The survey was hosted on Amazon Mechanical Turk (AMT), a crowdsourcing platform. AMT pays its workers, called “Turkers,” for completing micro-tasks, such as image annotation, or to answer a survey. Researchers buy credits on AMT, enabling a set number of tasks to be completed. AMT will advertise the task on behalf of the researcher and its Turkers will sign up to complete the tasks. Researchers are able to approve or reject the tasks based on the Turker’s performance. No identifiable information is passed through AMT; however, researchers could ask for identifiable information in surveys.

Turkers were informed that this survey was for a research study, how and when they could withdraw their data, that their participation should be voluntary, as well as any perceived risks and benefits to the research. They were provided with the email address of the lead author, should they have further questions about the survey. Ethical approval was obtained through the Usher Institute of Population Health Sciences and Informatics and through the Moray School of Education, both at the University of Edinburgh, and the survey abided by the Guidelines for Academic Requesters.

Turkers were paid US$ 1.75 for each completed survey, which we allotted 30 minutes for. As AMT ‘times out’ and participants would lose the ability to be reimbursed for their participation after a set time, we allotted much more than the time expected, as we did not want the survey to time out on any participants. The average time to complete the surveys was just under 7 minutes per survey.

### Questionnaire

CHNRI criteria were transformed into question statements (Appendix S1 in **Online Supplementary Document**). The criteria can also represent what one values more when investing into health research. For example, if there are competing interests, is it more important that one invests in research that reduces disease burden (criteria: disease burden reduction) or that is respectful to other cultures (acceptability/issues surrounding use)? The criteria and the corresponding questions can be found in **Table 1**.

**Table 1:** Criteria and corresponding questions asked

Criterion	Question
Equity	How important is it for the research to help health access become fairer between people?
Disease burden reduction	How important is it for the research to result in less disease? For example, if researchers were studying heart disease, could they reduce people having heart attacks?
Answerability	How important is it for the researchers to be able to create a study to properly answer their research question?
Effectiveness	How important is it that the results of the research have an impact and will people (including doctors, nurses, and patients) actually use them?
Deliverability	How important is it that the results of the research are affordable to those who need them and to those who pay for the results (for example, the national or local government, or patients)?
Feasibility	How important is it for the researchers to have enough time, funding and skilled staff to carry out the research?
Likelihood to fill a knowledge gap	How important is it for this research to result in new information?
Cost	How important is it for the results of this research to be less expensive than similar alternatives currently available? For example, if the research is looking at a drug for blood pressure, will the new drug be less expensive than the ones available now?
Sustainability	How important is it for the results to be long-lasting?
Acceptability/Issues surrounding use	How important is it for the research and the results of the research to be respectful to local beliefs and cultural practices?
Scale	How important is it that the results of the research will be widely available (for example, the results will be available throughout the country)?
Likelihood to attract national policy attention/Translational value	How important is it that the results of this research eventually turn into policy? For example, if a research is looking into a better way to identify diabetes, the government adopts the results and uses them to find people who have diabetes.
Implementation	How important is it that the intervention or results of this research can be changed to fit different groups of people (for example, different countries, regions in countries, or religions)? For example, medications that have cow-based products cannot be used in Hindu populations because of religious reasons – is it important for medicines not to use cow-based products?
Technical possibility	How important is it that if the research involves technology, that the technology is easy to use and not expensive to develop?
Innovation	How important is it that the research is trying to make something better than what is currently being used?

Non-identifiable individual and demographic information, such as age, gender, self-reported urban vs rural status, self-reported health status, country of residence, political views, immigration status, employment status and ethnicity were asked. The full survey is available in Appendix S1 in **Online Supplementary Document**.

Surveys were released in batches at different times of day to provide opportunities for Turkers living in different time-zones to answer and to facilitate a more global response. Location blocking was also used to facilitate as much of a global response as possible; in particular, as participants from India and the US were overrepresented in earlier surveys, in later surveys, “location blockers” were applied to these regions in order to encourage representation from other geographic regions. While AMT states that users from these countries cannot participate with their location-blocking function, users from these countries did participate in the ‘location-blocked’ surveys.

Questions to identify malicious Turkers, which are those who indiscriminately click on answers without reading questions, were included in the survey. Those who were identified as a malicious Turker were rejected from the study, their data was disposed of and excluded from the analysis. An example of a question to identify malicious Turkers is “please select the fourth star.” Further questions are available in Appendix S1 of **Online Supplementary Appendix.** 25 malicious Turkers (2% of respondents) were identified and their responses were excluded from the analysis.

### Data analysis

Descriptive statistics were calculated for individual and demographic variables. Due to low responses in certain categories, several categories of the variables were combined in order to prepare the data for random-intercept analysis. Black African, Black Caribbean, and “other black” ethnicities were combined into “Black,” under ethnicity, and Southeast and East Asian were combined into a joint category, due to a low number of respondents in the categories. Within marital status, separated, divorced, and widowed were combined into a new category of “no longer married,” while married, in a domestic partnership or a common-law relationship were combined into “married or in a domestic partnership/co-habiting.” Buddhist, Greek or Russian Orthodox, Mormon, and spiritual were added to the “other” religion category. Catholic and Christian denominations (including Protestant, Baptist, Lutheran, and Methodist) were combined into “Christian or Catholic.” In employment, not employed, disabled, not able to work, and retired were combined into a category of “not currently working.” Primary and secondary education were combined into “no higher education,” while completed college, university, graduate, or professional categories were combined into “higher education.” Non-binary and other genders were combined into “non-binary or other.” Finally, country of birth and country of residence were compared and used to create a proxy for immigration status; if country of residence was different than country of birth, immigration status was coded as “yes,” and if they were the same, it was coded as “no.” Additionally, countries were organised into the seven World Bank regions (Latin America and the Caribbean, North America, Europe and Central Asia, East Asia and the Pacific, South Asia, Middle East and North Africa, and Sub-Saharan Africa). Political beliefs, household size, age, and self-reported health status were treated as continuous variables in the analysis. For political beliefs, participants answered on a scale of 1-7 (extremely liberal to extremely conservative), which was used to code as a continuous variable. In self-reported health status, Likert scale responses were used to create a continuous variable.

Fixed intercept and random intercept models for each CHNRI criterion were compared to determine suitability for a random-intercept linear mixed effects model. This determined that a random-intercept linear mixed effects model was suitable in thirteen of fifteen cases. In order to account for variation between countries, the country of residence determined the random-intercept in the models. No random slopes were introduced to the models. In the remaining two cases, logistic regression was used to explore the relationship between the individual/demographic characteristics and the CHNRI criteria.

A forced-entry method was used for each random-intercept model using the maximum likelihood method, and the Akaike Information Criteria (AIC), Bayesian Information Criteria (BIC) and log likelihood ratio were used to determine goodness of fit.

In the logistic regression models, because the data was extremely left skewed, the Likert scale options for “very important” and “important” were combined to represent a positive response and the options for “neutral,” “slightly important,” and “not important at all’ were combined to represent a negative response. A forced entry method was used to build the models. The AIC, null and residual deviances were used to examine model fit. A model χ^2^ statistic determined that the model significantly predicted the fit better than a null model (χ^2^=50.66, df=25, *P=*0.002 for ‘acceptability’, and χ^2^=45.31, df=13, *P*<0.001 for ‘deliverability’). The Hosmer-Lemeshow and Nagelkerke’s goodness of fit tests were non-significant for both models, indicating acceptable fit in each case. There was no multicollinearity in either model.

Nonlinearity of continuous variables were tested against each outcome using multiple fractional polynomials. Several transformations were required due to nonlinearity for each of the four continuous variables. A legend displaying the transformations can be found in **Table 2****.**

**Table 2:** Legend and description of transformed variables

Category	Name of Transformed Variable	Description of transformation
Age	A1	Tenth of age
Household Size	H1	Reciprocal of a tenth of household size
	H2	Square root of a tenth of household size
	H3	Negative of square of tenth of household size
	H4	Log of a tenth of household size
Political beliefs	P1	Square root of a tenth of political affiliation
	P2	Log of a tenth of political affiliation
	P3	Negative of the square of tenth of political affiliation
	P4	Log of a tenth of political affiliation squared
Health status	HS1	Tenth of health status
	HS2	A tenth of health status cubed
	HS3	Negative of a tenth of health status squared
	HS4	Log of a tenth of health status
	HS5	Negative of a tenth of health status squared multiplied by the log of a tenth of health status

All analyses were completed in R Studio with R version 3.3.0 (R Studio, Boston, MA, USA).

## RESULTS

A total of 1031 Turkers from 73 countries, representing the 7 World Bank regions completed the survey. A summary of the individual and demographic characteristics can be found in **Table 3**.

**Table 3:** Individual and demographic characteristics of participants

Variable	Category	Number of participants (%)
Residence in World Bank Regions	Latin America & the Caribbean	133 (12.90)
	North America	330 (32.01)
	Europe & Central Asia	193 (18.72)
	East Asia & the Pacific	70 (6.79)
	South Asia	249 (24.15)
	Middle East & North Africa	24 (2.33)
	Sub-Saharan Africa	32 (3.10)
Born in World Bank Regions	Latin America & the Caribbean	143 (13.87)
	North America	296 (28.71)
	Europe & Central Asia	199 (19.30)
	East Asia & the Pacific	68 (6.60)
	South Asia	261 (25.32)
	Middle East & North Africa	32 (3.10)
	Sub-Saharan Africa	32 (3.10)
Immigration Status	Immigrated – Yes	126 (12.22)
	Immigrated – No	905 (87.79)
Urban v. Rural	Urban	753 (73.04)
	Rural	278 (26.96)
Ethnicity	Black (African, Caribbean, or Other)	64 (6.21)
	Central/South American	87 (8.44)
	South Asian	230 (22.31)
	Southeast and East Asian	122 (11.83)
	Middle Eastern	24 (2.33)
	White	481 (46.65)
	Multiple ethnicities	23 (2.23)
Marital Status	Married or in a domestic partnership/co-habiting	558 (54.12)
	No longer married (includes separated, divorced, widowed)	38 (3.69)
	Single	435 (42.19)
Religion	Atheist or agnostic	263 (25.51)
	Catholic or Christian	349 (33.85)
	Jewish	9 (0.87)
	Hindu	188 (18.23)
	Muslim	83 (8.05)
	Other	139 (13.48)
Employment	Employed, working full-time	607 (58.87)
	Employed, working part-time	126 (12.22)
	Self-employed	120 (1.16)
	Student	91 (8.83)
	Not currently working (including not employed, disabled, and retired)	87 (8.44)
Health Stakeholder	Yes	269 (26.09)
	No	762 (73.91)
Education	No higher education	68 (6.59)
	Some college, but no degree	145 (14.06)
	Higher education	818 (79.34)
Political beliefs	Extremely liberal	102 (9.89)
	Moderately liberal	288 (27.93)
	Slightly liberal	169 (16.39)
	Neither liberal nor conservative	243 (23.57)
	Slightly conservative	111 (10.77)
	Moderately conservative	79 (7.66)
	Extremely conservative	39 (3.78)
Self-reported health status	Excellent	187 (18.14)
	Good	608 (58.97)
	Neutral	183 (17.75)
	Poor	45 (4.36)
	Very poor	8 (0.78)
Gender	Male	675 (65.47)
	Female	350 (33.95)
	Non-binary or other	6 (0.58)
Age	Minimum	18
	Maximum	70
	IQR	25.50 to 36.00
	Mean	31.85
Household size	Minimum	1.0
	Maximum	12.0
	IQR	2.0 to 4.5
	Mean	3.50

IQR – interquartile range

**Table 4** displays the b-values and confidence intervals for all random-intercept models, with p-values indicated. Each model is displayed within a column, with estimates given in the corresponding rows. Rows without estimates were not including in the respective models, due to model fit. A legend of the transformations can be found in **Table 2**. **Table 5** contains the results of the logistic regression models. **Table 6** displays a summary of the individual and demographic characteristics and the criteria they differ in.

**Table 4:** Results of random-intercept models (b-value and 95% confidence intervals)

Individual and demographic characteristics		Equity	Disease burden reduction	Effectiveness	Feasibility	Likelihood to fill a gap	Cost	Sustainability	Acceptability	Scale	Implementation	Translational value	Technical possibility	Innovation
Model Intercept		4.09 (3.85 to 4.33)***	3.80 (3.48 to 4.11)***	3.6 2 (3.32 to 3.92)***	3.51 (3.00 to 4.03)***	3.93 (3.78 to 4.08)***	3.50 (3.12 to 3.89)***	3.89 (3.62 to 4.15)***	2.07 (1.67 to 2.47)***	3.52 (3.21 to 3.83)***	-3.88 (-7.09 to -0.68)*	3.83 (3.54 to 4.12)***	3.83 (3.64 to 4.02)***	4.65 (4.29 to 5.02)***
Immigration Status	No (Ref)	–	–	–	–	–	–	–	–	–	–	–	–	–
	Yes	0.13 (-0.01 to 0.28)	–	–	–	–	–	–	–	–	–	0.24 (0.05 to 0.45)**	–	–
Ethnicity	White (Ref)	–	–	–	–	–	–	–	–	–	–	–	–	–
	Black	0.32 (0.12 to 0.52)**	0.13 (-0.07 to 0.33)	0.32 (0.12 to 0.51)**	0.23 (0.05 to 0.41)**	–	0.46 (0.18 to 0.74)**	0.36 (0.10 to 0.61)**	0.07 (-0.24 to 0.38)	–	0.34 (0.02 to 0.66)*	–	0.55 (0.26 to 0.85)***	–
	Central/ South American	0.10 (-0.09 to 0.29)	-0.22 (-0.39 to -0.05)**	0.07 (-0.12 to 0.25)	-0.01 (-0.17 to 0.16)	–	-0.03 (-0.29 to 0.23)	0.12 (0.11 to 0.36)	0.002 (-0.27 to 0.27)	–	-0.32 (-0.59 to -0.04)*	–	0.22 (-0.05 to 0.49)	–
	South Asian	0.11 (-0.05 to 0.27)	-0.11 (-0.28 to 0.07)	-0.02 (-0.19 to 0.14)	0.13 (-0.04 to 0.30)	–	0.12 (-0.14 to 0.38)	0.24 (0.03 to 0.46)*	0.33 (0.05 to 0.61)*	–	0.12 (-0.17 to 0.40)	–	0.10 (-0.18 to 0.38)	–
	Southeast and East Asian	0.26 (0.09 to 0.42)**	-0.09 (-0.25 to 0.07)	-0.002 (-0.16 to 0.16)	0.10 (-0.05 to 0.26)	–	0.19 (-0.04 to 0.43)	0.22 (0.01 to 0.43)*	0.18 (-0.07 to 0.43)	–	-0.15 (-0.41 to 0.11)	–	0.06 (-0.20 to 0.31)	–
	Middle Eastern	0.27 (-0.05 to 0.58)	-0.32 (-0.67 to 0.02)	-0.21 (-0.52 to 0.09)	-0.02 (-0.33 to 0.28)	–	-0.30 (-0.76 to 0.17)	-0.26 (-0.64 to 0.13)	0.60 (0.07 to 1.14)*	–	-0.30 (-0.85 to 0.25)	–	-0.04 (-0.53 to 0.45)	–
	Multiple ethnicity	0.34 (0.03 to 0.65)*	-0.12 (-0.43 – 0.19)	-0.10 (-0.40 to 0.19)	-0.23 (-0.50 to 0.04)	–	-0.03 (-0.45 to -0.38)***	-0.02 (-0.39 to 0.36)	-0.11 (-0.60 to 0.37)	–	0.12 (-0.37 to 0.62)	–	0.42 (-0.02 to 0.84)	–
Marital Status	Married (Ref)	–	–	–	–	–	–	–	–	–	–	–	–	–
	No longer married†	–	–	–	–	–	–	-0.19 (-0.49 to 0.11)	-0.02 (-0.40 to 0.37)	–	–	–	–	–
	Single	–	–	–	–	–	–	-0.24 (-0.36 to -0.12)***	-0.24 (-0.40 to -0.09)**	–	–	–	–	–
Religion	Atheist/ agnostic (Ref)	–	–	–	–	–	–	–	–	–	–	–	–	–
	Catholic/ Christian	–	0.03 (-0.10 to 0.15)	–	-0.02 (-0.14 to 0.09)	–	0.21 (0.04 to 0.38)*	–	0.49 (0.29 to 0.69)***	–	0.41 (0.20 to 0.61)***	0.26 (0.09 to 0.43)**	0.17 (-0.004 to 0.35)	–
	Jewish	–	-0.02 (-0.51 to 0.48)	–	-0.15 (-0.59 to 0.29)	–	0.27 (-0.38 to 0.93)	–	0.07 (-0.70 to 0.85)	–	0.46 (-0.33 to 1.25)	0.62 (-0.07 to 1.31)	0.03 (-0.46 to 0.91)	–
	Hindu	–	-0.21 (-0.41 to -0.002)*	–	-0.29 (-0.48 to -0.09)**	–	0.34 (0.05 to 0.63)*	–	0.66 (0.34 to 0.98)***	–	0.77 (0.44 to 1.10)***	0.54 (0.27 to 0.82)***	0.42 (0.11 to 0.73)**	–
	Muslim	–	0.04 (-0.18 to 0.27)	–	-0.03 (-0.24 to 0.18)	–	0.25 (-0.06 to 0.57)	–	0.60 (0.24 to 0.95)**	–	0.81 (0.43 to 1.18)***	0.67 (0.39 to 0.95)***	0.28 (-0.05 to 0.61)	–
	Other	–	-0.03 (-0.19 to 0.12)	–	0.06 (-0.07 to 0.02)	–	0.13 (-0.07 – 0.34)	–	0.31 (0.07 to 0.55)**	–	0.34 (0.09 to 0.58)**	0.28 (0.07 to 0.50)**	0.16 (-0.05 to 0.38)	–
Employment	Full-time (Ref)	–	–	–	–	–	–	–	–	–	–	–	–	–
	Part-time	-0.16 (-0.31 to -0.02)*	-0.15 (-0.29 to -0.01)*	–	–	-0.09 (-0.26 to 0.08)	-0.10 (-0.29 to 0.10)	–	-0.23 (-0. to -0.004)*	0.06 (-0.09 to 0.21)	–	-0.09 (-0.29 to 0.11)	-0.07 (-0.27 to 0.13)	–
	Self-employed	0.07 (-0.08 to 0.21)	-0.001 (-0.15 to 0.15)	–	–	-0.16 (-0.33 to 0.01)	-0.19 (-0.38 to 0.01)	–	-0.08 (-0.31 to 0.15)	0.17 (0.02 to 0.33)*	–	-0.03 (-0.24 to 0.17)	-0.21 (-0.41 to -0.01)*	–
	Student	0.01 (-0.17 to 0.19)	-0.02 (-0.20 – 0.15)	–	–	-0.21 (-0.41 to -0.01)*	-0.21 (-0.44 to 0.01)	–	0.01 (-0.26 to 0.28)	0.10 (-0.09 to 0.29)	–	-0.08 (-0.32 to 0.15)	-0.38 (-0.61 to -0.14)**	–
	Not working	0.03 (-0.14 to 0.20)	-0.02 (-0.19 to 0.15)	–	–	-0.20 (-0.39 to 0.004)	-0.23 (-0.46 to -0.01)*	–	-0.20 (-0.47 to 0.07)	0.11 (-0.07 to 0.29)	–	-0.25 (-0.59 to -0.11)**	-0.14 (-0.38 to 0.09)	–
Health stakeholder	No (Ref)	–	–	–	–	–	–	–	–	–	–	–	–	–
	Yes	–	-0.18 (-0.29 to -0.06)**	-0.18 (-0.29 to -0.07)***	-0.17 (-0.27 to -0.08)***	–	–	–	0.34 (0.17 to 0.52)***	–	0.19 (0.02 to 0.37)*	–	–	-0.12 (-0.24 to -0.01)*
Education	No higher education (Ref)	–	–	–	–	–	–	–	–	–	–	–	–	–
	Some college	–	–	–	–	–	–	–	–	0.28 (0.06 to 0.52)*	–	–	–	–
	Higher education	–	–	–	–	–	–	–	–	0.11 (-0.09 to 0.32)	–	–	–	–
Gender	Male (Ref)	–	–	–	–	–	–	–	–	–	–	–	–	–
	Female	–	0.11 (0.01 to 0.21)*	0.17 (0.08 to 0.27)***	0.14 (0.05 to 0.22)**	0.21 (0.10 to 0.33)***	0.16 (0.03 to 0.29)**	0.13 (0.01 to 0.24)*	0.33 (0.18 to 0.48)***	–	0.18 (0.02 to 0.33)*	–	–	–
	Non-binary/ Other	–	0.08 (-0.52 to 0.67)	0.40 (-0.20 – 0.96)	0.44 (-0.08 to 0.97)	0.34 (-0.36 to 1.04)	-0.01 (-0.80 to 0.79)	0.09 (-0.62 to 0.81)	-0.31 (-1.25 to 0.63)	–	-0.92 (-1.88 to 0.04)	–	–	–
Political beliefs	Linear continuous	-0.05 (-0.08 to -0.02)***	–	–	–	–	–	–	–	–	–	–	–	–
	P2	–	–	–	–	–	–	–	–	–	7.60 (3.98 to 11.22)***	–	–	–
	P3	–	–	–	–	–	–	–	–	–	-2.19 (-3.15 to -1.24)***	–	–	0.76 (0.35 to 1.17)***
	P4	–	–	–	–	–	–	–	–	0.003 (0.001 to 0.005)**	–	–	–	–
	P6	–	–	–	–	–	–	–	–	–	–	–	–	0.30 (0.15 to 0.45)***
Self-reported health status	Linear continuous	–	0.08 (0.02 to 0.14)**	–	–	–	0.78 (-0.02 to 1.58)	–	0.13 (0.04 to 0.23)**	–	–	–	–	0.06 (0.002 to 0.12)*
	Health status 1	–	–	1.23 (0.66 to 1.81)***	–	–	–	–	–	–	–	–	–	–
	Health status 2	2.53 (1.13 to 3.92)***	–	–	3.92 (1.49 to 6.34)*	2.47 (0.83 to 4.12)**	–	–	–	3.23 (1.76 to 4.71)***	–	–	–	–
	Health status 3	–	–	–	–	–	–	–	–	–	–	-0.10 (-0.16 to -0.04)***	–	–
	Health status 4	–	–	–	-0.35 (-0.69 to -0.02)**	–	–	–	–	–	–	–	–	–
	Health status 5	–	–	–	–	–	–	–	–	–	–	-0.04 (-0.07 to -0.02)***	–	–
Age	Linear continuous	0.01 (0.001 to 0.01)**	–	–	–	–	–	0.01 (0.00 to 0.01)*	–	–	–	–	–	–
	Age 1	–	1.23 (0.76 to 1.88)*	0.85 (0.34 to 1.35)***	0.49 (0.03 to 0.96)*	–	–	–	–	1.33 (0.75 to 1.90)***	–	–	–	–
Household Size	Linear continuous	–	–	–	–	–	–	–	–	–	–	–	–	–
	Household size 2	–	–	–	–	–	-0.06 (-0.08 to -0.03)***	–	–	–	–	–	–	–
	Household size 3	–	–	–	–	–	–	–	–	–	-0.20 (-0.35 to -0.05)**	–	–	–
	Household size 4	–	–	–	-0.01 (-0.01 to -0.002)***	–	–	–	–	–	–	–	-0.004 (-0.01 to -0.002)***	-0.003 (-0.005 to -0.001)***
	Household size 5	–	–	–	-0.24 (-0.41 to -0.08)**	–	–	–	–	–	–	–	–	–

Table displays thirteen random-intercept models. Each model is displayed in a column, with the dependent variable listed at the head of the column, and the independent variables listed within each row. Where there is a “–“ in a cell, the variable was not included in the model due to impacted the fit negatively. Each cell displays the b-value and 95% confidence intervals. *P*-values are denoted as follows: *0.05, *0.01, ***0.001.

†No longer married includes participants who are separated.

**Table 5:** Results of logistic regression analyses exploring characteristics of respondents and relationship to CHNRI criteria for health research prioritization

Demographic Characteristics	Category	Odds ratio	95% confidence interval		*P*-value	
**Lower**	**Upper**
**Answerability**						
Intercept	N/A	2.87	0.98	8.51	0.06	
World Bank region	Reference – North America	–	–	–	–	0.02
	Europe & Central Asia	1.49	0.84	2.69	0.18	
	East Asia & the Pacific	9.29	1.29	195.29	0.06	
	South Asia	0.75	0.21	2.36	0.63	
	Middle East & North Africa	3.08	0.50	27.31	0.25	
	Sub-Saharan Africa	4.84	0.74	95.84	0.16	
	Latin America & the Caribbean	1.65	0.72	4.07	0.25	
Ethnicity	Reference – White	–	–	–	–	0.58
	Central/South American	2.32	0.81	7.75	0.14	
	East Asian	3.06	0.81	20.32	0.15	
	South Asian	2.93	0.94	9.61	0.07	
	Southeast Asian	1.82	0.48	7.68	0.39	
	Middle Eastern	1.39	0.31	8.09	0.69	
	Black	1.05	0.40	3.30	0.93	
	Multiple Ethnicity	1.22	0.33	7.96	0.80	
Religion	Reference – Atheist/Agnostic	–	–	–	–	0.10
	Catholic	0.66	0.33	1.33	0.24	
	Christian	0.76	0.39	1.49	0.41	
	Hindu	0.75	0.26	2.10	0.60	
	Muslim	0.45	0.16	1.32	0.14	
	Spiritual/non-religious	2.70	0.91	11.64	0.11	
	Other	0.47	0.22	1.08	0.06	
Self-reported health status	Reference – Excellent	–	–	–	–	0.12
	Good	0.73	0.38	1.31	0.31	
	Neutral	0.44	0.22	0.86	0.02	
	Poor	0.81	0.28	2.69	0.71	
Gender	Reference – Male	–	–	–	–	0.07
	Female	1.70	1.05	2.83	0.04	
	Non-binary/other	0.39	0.05	8.23	0.43	
Age	(continuous)	1.03	1.01	1.06	0.02	0.01
Deliverability						
Intercept	N/A	2.23	0.88	5.74	0.09	
World Bank regions	Reference – North American	–	–	–	–	0.06
	Europe & Central Asia	1.88	1.06	3.44	0.03	
	East Asia & the Pacific	2.10	0.91	5.76	0.11	
	South Asia	0.94	0.59	1.52	0.81	
	Middle East & North Africa	2.15	0.59	13.89	0.32	
	Sub-Saharan Africa	4.13	0.84	74.89	0.17	
	Latin America & the Caribbean	0.96	0.55	1.74	0.90	
Immigration Status	Reference – No	–	–	–	–	0.02
	Yes	2.41	1.19	5.55	0.02	
Self-reported Health Status	Reference – Excellent	–	–	–	–	0.01
	Good	0.77	0.43	1.31	0.35	
	Neutral	0.39	0.21	0.72	0.003	
	Poor	0.43	0.18	1.06	0.06	
Gender	Reference – Male	–	–	–	–	0.001
	Female	1.60	1.06	2.46	0.03	
	Non-binary/other	1.00	0.14	19.64	0.98	
Age	(Continuous)	1.04	1.01	1.06	0.01	0.08

**Table 6:** Summary of demographic characteristics and criteria in which there are significant differences found.

Demographic Characteristic	List of criteria where there are significant differences	Total (N)
Self-reported health status	Equity	11
	Disease burden reduction	
	Effectiveness	
	Feasibility	
	Likelihood to fill a knowledge gap	
	Acceptability	
	Scale	
	Innovation	
	Translational value	
	Answerability	
	Deliverability	
Gender	Disease burden reduction	10
	Effectiveness	
	Feasibility	
	Likelihood to fill a knowledge gap	
	Cost	
	Sustainability	
	Acceptability	
	Implementation	
	Answerability	
	Deliverability	
Ethnicity	Equity	9
	Disease burden reduction	
	Effectiveness	
	Feasibility	
	Cost	
	Sustainability	
	Acceptability	
	Implementation	
	Technical possibility	
Employment	Equity	8
	Disease burden reduction	
	Likelihood to fill a knowledge gap	
	Cost	
	Acceptability	
	Scale	
	Translational value	
	Technical possibility	
Religion	Disease burden reduction	7
	Feasibility	
	Cost	
	Acceptability	
	Implementation	
	Translational value	
	Technical possibility	
Age	Equity	7
	Disease burden reduction	
	Effectiveness	
	Feasibility	
	Scale	
	Answerability	
	Deliverability	
Health stakeholder	Disease burden reduction	6
	Effectiveness	
	Feasibility	
	Acceptability	
	Implementation	
	Innovation	
Household size	Feasibility	5
	Cost	
	Implementation	
	Technical possibility	
	Innovation	
Political views	Equity	4
	Scale	
	Implementation	
	Innovation	
Immigration status	Translational value	2
	Deliverability	
Marital status	Sustainability	2
	Acceptability	
Education	Scale	1

Results are discussed below, by independent variable, across models. Results are presented in beta-values unless otherwise specified.

### Immigration

Compared to those who are living in their country of birth, those classified as immigrants only differed from those who weren’t on two of fifteen criteria. Those who have immigrated find the potential for research to translate to policy more important (0.24, confidence interval 95% 95% CI = 0.05 to 0.45, *P* = 0.01) and were more likely to find deliverability to be important than those not classified as immigrants in a logistic regression (OR = 2.41, 95% 95% CI = 1.19 to 5.55, *P* = 0.02).

### Ethnicity

The largest differences in ethnicity were found between black and white Turkers. Compared to white Turkers, Black Turkers rated equity (0.32, 95% CI = 0.12 to 0.52, *P* = 0.002), effectiveness (0.32, 95% CI = 0.12 to 0.51, *P* = 0.002), feasibility (0.23, 95% CI = 0.12 to 0.41, *P* = 0.01), cost (0.46, 95% CI = 0.18 to 0.74, *P* = 0.002), sustainability (0.36, 95% CI = 0.10 to 0.61, *P* = 0.01), implementation (0.34, 95% CI = 0.02 to 0.66, *P* = 0.04), and technical possibility (0.55, 95% CI = 0.26 to 0.85, *P* < 0.001) significantly more important. Middle Eastern Turkers were the least different from White Turkers, only differing on the acceptability criterion, which they rated significantly more important than white Turkers (0.60, 95% CI = 0.07 to 1.14, *P* = 0.03). South Asians similarly ranked acceptability more important than white Turkers (0.33, 95% CI = 0.05 to 0.61, *P* = 0.02). Those who were Central or South American rated burden reduction (-0.22, 95% CI = -0.39 to -0.05, *P* = 0.01) and implementation (-0.32, 95% CI = -0.59 to -0.04, *P* = 0.03) significantly less important than white Turkers. South, Southeast and East Asians rated sustainability more important (0.24, 95% CI = 0.03 to 0.46, *P* = 0.02; 0.22, 95% CI = 0.01 to 0.43, *P* = 0.04, respectively). Finally, those who identified as being multi-ethnic rated cost less important (-0.02, 95% CI = -0.45 to -0.38) and equity more important (0.34, 95% CI = 0.03 to 0.65, *P* = 0.03) compared to white Turkers.

### Marital status

Single Turkers found sustainability and acceptability significantly less important than married Turkers (-0.24, 95% CI = -0.36 to -0.12, *P* < 0.001; -0.24, 95% CI = -0.24, CI-0.24 to -0.09, *P* = 0.003, respectively).

### Religion

Compared to those who were atheist or agnostic, those who were Hindu differed the most in their valuation of CHNRI criteria. All religions, compared to those who were atheist or agnostic, attributed greater significance to the acceptability (Christian/Catholic, 0.49, 95% CI = 0.29 to 0.69, *P* < 0.001; Hindu, 0.66, 95% CI = 0.34 to 0.98, *P* < 0.001; Muslim, 0.60, 95% CI = 0.24 to 0.96, *P* = 0.001; ‘Other,’ 0.31, 95% CI = 0.07 to 0.55, *P* = 0.01), implementation (Christian/Catholic, 0.41, 95% CI = 0.20 to 0.61, *P* < 0.001; Hindu, 0.77, 95% CI = 0.44 to 1.10, *P* < 0.001; Muslim, 0.81, 95% CI = 0.43 to 1.18, *P* < 0.001; “Other,” 0.34, CI 0.09 to 0.58, *P* = 0.01), and translational value (Christian/Catholic, 0.26, 95% CI = 0.09 to 0.43, *P* = 0.003; Hindu, 0.54, 95% CI = 0.27 to 0.82, *P* < 0.001; Muslim, 0.67, 95% CI = 0.39 to 0.95, *P* < 0.001; ‘Other,’ 0.28, 95% CI = 0.07 to 0.50, *P* = 0.01).

Those who were Hindu also ranked cost (0.34, 95% CI = 0.05 to 0.63, *P* = 0.02), and technical possibility (0.42, 95% CI = 0.11 to 0.73, *P* = 0.01) higher than those who were atheist or agnostic. Conversely, Hindu Turkers ranked disease burden reduction and feasibility less important than those who were atheist or agnostic (-0.21, 95% CI = -0.41 to -0.002, P = 0.05; -0.29, 95% CI = -0.48 to -0.09, P = 0.004, respectively). Catholic or Christian Turkers also rated cost (0.21, 95% CI = 0.04 to 0.38, *P* = 0.02) higher than those who were atheist or agnostic. There were no significant differences between Turkers who were Jewish to those who were atheist or agnostic, though the sample size was low and there may not have been sufficient power to detect this difference.

### Employment

Compared to those employed full-time, those employed part-time found equity (-0.16, 95% CI = -0.31 to -0.02, *P* = 0.03), disease burden reduction (-0.15, 95% CI = -0.29 to -0.01, *P* = 0.04), and acceptability (-0.23, 95% CI = -0.45 to -0.004, *P* = 0.05) less important. Those who were self-employed found technical possibility less important (-0.21, 95% CI = -0.41 to -0.01, *P* = 0.03), but found scale (-0.17, 95% CI = 0.02 to 0.33, *P* = 0.03) more important. Students found the likelihood of the research to fill a knowledge gap less important (-0.21, CI -0.41 to -0.01, *P* = 0.002) as well as technical possibility of conducting the research (-0.38, 95% CI = -0.61 to -0.14, *P* = 0.04) compared to those employed full-time. Those currently not working found cost (-0.23, 95% CI = -0.46 to -0.01, *P* = 0.05) and translational value (-0.25, 95% CI = -0.59 to -0.11, *P* = 0.004) to be less important than those working full-time.

### Health stakeholder

Those who identified as being a health stakeholder found disease burden reduction (-0.18, 95% CI = -0.29 to -0.06, *P* = 0.002), effectiveness (-0.18, 95% CI = -0.29 to -0.07, *P* = 0.001), feasibility (-0.17, 95% CI = -0.27 to -0.08, *P* = 0.001) and innovation (-0.12, 95% CI = -0.24 to -0.01, *P* = 0.03) to be less important than those who did not identify as health stakeholders. Conversely, those who identified as health stakeholders found acceptability (0.34, 95% CI = 0.17 to 0.52, *P* < 0.001) and implementation (0.19, 95% CI = 0.02 to 0.37, *P* = 0.03) to be more important than those who did not identify as health stakeholders.

### Education

Education only had a significant effect on scale, with those who enrolled, but did not complete, a college degree finding scale to be more important than those with no higher education (0.28, 95% CI = 0.06 to 0.52, *P* = 0.01).

### Gender

Gender was one of the most important attributes regarding differences in perceived important of CHNRI criteria, with significant differences in 10 of 15 (2/3) of criteria. In each model where gender was included in the model, being female was a predictor for finding the criterion important in comparison to being male. The models in which gender was a predictor were: disease burden reduction (0.11, 95% CI = 0.01 to 0.21, *P* = 0.03), effectiveness (0.17, 95% CI = 0.08 to 0.27, *P* < 0.001), feasibility (0.14, 95% CI = 0.05 to 0.22, *P* = 0.002), likelihood to fill a knowledge gap (0.21, 95% CI = 0.10 to 0.33, *P* < 0.001), cost (0.16, 95% CI = 0.03 to 0.29, *P* = 0.02), sustainability (0.13, 95% CI = 0.01 to 0.24, *P* = 0.03), acceptability (0.22, 95% CI = 0.18 to 0.48, *P* < 0.001), and implementation (0.18, 95% CI = 0.02 to 0.33, *P* = 0.03). In the logistic regression model, female respondents were more likely to find deliverability more important compared to male respondents (OR = 1.60, 95% CI = 1.06 to 2.46, *P* = 0.03).

### Political beliefs

Increasing conservatism was negatively associated with equity (-0.05, 95% CI = -0.08 to -0.02, *P* = 0.0003). Several nonlinear transformations of political affiliation were imputed. P1 was positively associated with implementation (7.60, 9.98 to 11.22, *P* < 0.001). P2 was negatively associated with implementation (-2.19, 95% CI = -3.15 to -1.24, *P* < 0.001) and was positively associated with innovation (0.76, 95% CI = 0.35 to 1.17, *P* < 0.001). P3 was positively associated with scale (0.003, 95% CI = 0.001 to 0.005, *P* = 0.002), and P4 was positively associated with innovation (0.30, 95% CI = 0.15 to 0.45, *P* < 0.001).

### Self-reported health status

The linear variable of self-reported health status was positively correlated with disease burden reduction (0.08, 95% CI = 0.02 to 0.14, *P* = 0.01), acceptability (0.13, 95% CI = 0.04 to 0.23, *P* = 0.01), and innovation (0.06, 95% CI = 0.002 to 0.12, *P* = 0.04). Several nonlinear transformations were imputed into the models, and were significantly correlated with the CHNRI criteria. HS1 was significantly positively correlated with effectiveness (1.23, 95% CI = 0.66 to 1.81, *P* < 0.001). HS2 was positively correlated with equity (2.53, 95% CI = 1.13 to 3.92, *P* = 0.0004), feasibility (3.92, 95% CI = 1.49 to 6.34, *P* = 0.002), likelihood to fill a knowledge gap (2.47, 95% CI = 0.83 to 4.12, *P* = 0.003), and scale (3.23, 1.76 to 4.71, *P* < 0.001). HS3 was negatively correlated with translational value (-0.10, 95% CI = -0.16 to -0.04, *P* = 0.001). HS4 was negatively correlated with feasibility (-0.35, 95% CI = -0.69 to -0.02, *P* = 0.04), and HS5 was negatively correlated with translational value (-0.04, 95% CI = -0.07 to -0.02, *P* = 0.001). In the logistic regression models, those who had a self-reported health status as neutral found answerability and deliverability significantly less important than those with an excellent health status (OR = 0.44, 95% CI = 0.22 to 0.86, *P* = 0.02; OR = 0.39, 95% CI = 0.21 to 0.72, *P* = 0.003, respectively).

### Age

Age as a linear variable was positively correlated with equity (0.01, 95% CI = 0.001 to 0.010, *P* = 0.01). As a transformation (A1), age was positively associated with disease burden reduction (1.12, 95% CI = 0.75 to 1.88, *P* < 0.001), effectiveness (0.85, 95% CI = 0.34 to 1.35, *P* = 0.001), feasibility (0.49, 95% CI = 0.02 to 0.96, *P* = 0.04), sustainability (0.01, 95% CI = 0.00 to 0.01, *P* = 0.05), and scale (1.33, 95% CI = 0.75 to 1.90, *P* < 0.001). Increasing age was a significant predictor of finding answerability and deliverability important in the logistic regression models (OR = 1.03, 95% CI = 1.01 to 1.06, *P* = 0.02; OR = 1.04, 95% CI = 1.01 to 1.06, *P* = 0.01, respectively).

### Household size

H1 was negatively associated with cost (-0.06, 95% CI = -0.08 to -0.03, *P* <  0.001), while H2 was negatively associated with implementation (-0.20, 95% CI = -0.35 to -0.05, *P* = 0.01). H3 was negatively associated with feasibility (-0.01, 95% CI = -0.01 to -0.002, *P* = 0.001), technical possibility (-0.004, 95% CI = -0.01 to -0.002, *P* < 0.001), and innovation (-0.003, 95% CI = -0.005 to -0.001, *P* < 0.001). H4 was negatively associated with feasibility (-0.24, 95% CI = -0.41 to -0.08, *P* = 0.005).

## DISCUSSION

The results show that within many of the criteria, there are differences in relative importance of criteria from responders. The individual and demographic characteristics that were most commonly associated with differences across criteria were self-reported health status, which was significantly associated with differences in responses across 11 criteria, gender, which was significantly associated with differences in responses across 10 criteria, ethnicity, which was significantly associated with differences in responses across 9 criteria, and, employment and religion, which were significantly associated with differences in responses across 8 and 7 criteria, respectively.

Disease burden reduction, feasibility, and acceptability had the most individual and demographic characteristics that contributed to differences in their perceived importance; each of these criteria had 7 individual or demographic characteristics that significantly contributed to their perceived importance. Demographic and individual characteristics were least predictive of responses in likelihood to fill a knowledge gap, answerability, and sustainability. Interestingly, disease burden reduction, feasibility, answerability, and sustainability all have relatively high mean scores (4.42, 4.41, and 4.40 respectively), which would limit the variation in responses. Acceptability had the lowest mean among all criteria (3.11), which may be reflective of heterogeneity of the Turkers.

There were several counterintuitive results. Having a larger household size was negatively correlated with being concerned with the cost of the product of the research; one may assume that having a large household would result in financial constraints and more concern for cost. Those who were unemployed were less likely to consider cost or translational value (ie, that the research would inform policy) important, in comparison to those employed full-time. Additionally, those who were classified as health stakeholders were less likely to rank disease burden reduction, effectiveness, feasibility, or innovation as important in comparison with those who were not. While these results are indeed counterintuitive, the data was extremely left skewed. The resulting patterns may be not that these groups do not find these criteria unimportant; rather, it may demonstrate that they simply find them slightly less important than their counterparts. However, it may be interesting to run the experiment again asking participants to allocate a truly relative valuation of the criteria, for example through allocation of imaginary money amongst the criteria.

There have been no CHNRI exercises that have involved stakeholder groups that have asked stakeholders information on their health status, gender, employment status, religion, or ethnicity to the authors’ knowledge. However, this information may be important in achieving a balanced, well-rounded and representative approach to forming a stakeholder group, especially one involving the public. Being female vs male was a significant predictor of finding 10 of the 15 criteria important. While many CHNRI exercises report on the gender of the researchers, none have reported on the gender of the stakeholder groups [6,8-11].

While no CHNRI exercises collected demographic data with regards to the stakeholder portion of the exercise, one asked health stakeholders (those working in national or district hospitals, health facilities, teaching hospitals, or in United Nations posts) in Uganda whether demographic characteristics of patients (eg, age, religion, societal power, affluence, mental, and physical capabilities) should be criteria to influence the priorities, but still did not report even gender-related information on the stakeholder group weighting the criteria [12].

Our data shows that self-reported health status was the most important predictor of differences within 11 of 15 criteria, more than any other demographic; this indicates that forming a stakeholder group of people affected by a disease may provide a unique perspective in terms of needs and values.

Being a health stakeholder, defined by responding yes to working or having worked in the health sector, was a predictor of a difference in rating 6 of the 15 CHNRI criteria, and all 5 of the original and most widely used CHNRI criteria. Many exercises that have employed stakeholder groups have used the original CHNRI criteria, but few have employed non-health stakeholders (eg, members of public, patients with the disease or condition, caregivers of patients, etc.). It may be important for future exercises to include these groups, as there are differences in how they view the importance of criteria. While researchers or health professionals may have a particular lens to viewing a criterion, a member of the public may find another aspect of research more of a priority and it can be important to consider this wider perspective as well.

### Limitations

This exercise explores the associations between individual and demographic characteristics and CHNRI criteria using data collected from AMT, which is a crowdsourcing platform. The Turkers who participated live in 73 countries have varying experiences with health and research, and varying knowledge of what health research is and could be. They also may have varying experiences with disease, which may be important. This group of stakeholders aims to be representative of a general public opinion, and not a targeted stakeholder group for a specific disease. It would be expected that results would be different for a stakeholder group with a specific condition. When designing CHNRI studies, including this type of stakeholder group should be considered.

Moreover, as with many crowdsourcing studies, there are concerns about the generalisability of the data. Since crowdsourcing surveys use ‘self-selected’ participants, their views may differ from those who would not opt to answer this type of survey. A previous study has shown that Turkers tend to be wealthier, younger, and better educated than participants of traditional survey research [13]. Moreover, Turkers must have access to technology as a prerequisite for participating in the study, as it is hosted online. This may make their experiences different from those without access to technology, or without knowledge of AMT, and the generalisability of the survey should be considered. Still, sampling views from over 1000 participants in over 70 countries in under two weeks is no small feat and would not be possible without access to crowdsourcing technology.

We were unable to model interactions in our data, or to include random slopes, due insufficient power. This could be explored in the future with more data, and with increased respondents in each category.

Finally, we collected data for household-level income based on country of origin, and hoped to standardize to income quintiles but were unable to conduct this analysis due to inability to access standardised data for all countries; thus, income was excluded as a variable. However, if efforts to obtain data on income by wealth quintiles for each country are successful, further exploration of the effect of income on the relative importance of CHNRI criteria is warranted.

## CONCLUSION

It will be imperative for CHNRI exercises in the future to collect basic individual or demographic information on the stakeholder groups in addition to the researcher groups contributing to health research prioritisation exercises. Not doing so risks a lack of transparency regarding possible significant biases in the relative importance of CHNRI criteria. Moreover, including diverse stakeholder groups, including non-health stakeholders and those affected by the condition being researched, may be important to achieving representation of different viewpoints, which is the intention of the criteria weighting step in CHNRI.

## Additional material

Online Supplementary Document

## References

[1] Rudan I, Gibson JL, Ameratunga S, El Arifeen S, Bhutta ZA, Black M (2008). Setting priorities in global child health research investments: guidelines for implementation of CHNRI method.. Croat Med J.

[2] Yoshida S, Wazny K, Cousens S, Chan KY (2016). Setting health research priorities using the CHNRI method: III. Involving stakeholders.. J Glob Health.

[3] Viergever RF, Olifson S, Ghaffar A, Terry RF (2010). A checklist for health research priority setting: nine common themes of good practice.. Health Res Policy Syst.

[4] Wazny K, Sadruddin S, Zipursky A, Hamer DH, Jacobs T, Kallander K (2014). Setting global research priorities for integrated community case management (iCCM): Results from a CHNRI (Child Health and Nutrition Research Initiative) exercise.. J Glob Health.

[5] Yoshida S, Cousens S, Wazny K, Chan KY (2016). Setting health research priorities using the CHNRI method: II. Involving researchers.. J Glob Health.

[6] Arora NK, Mohapatra A, Gopalan HS, Wazny K, Thavaraj V, Rasaily R (2017). Setting research priorities for maternal, newborn, child health and nutrition in India by engaging experts from 256 indigenous institutions contributing over 4000 research ideas: a CHNRI exercise by ICMR and INCLEN.. J Glob Health.

[7] Wazny K Ravenscroft J Chan K Bassani D Anderson N Rudan I Setting global and regional weights for fifteen CHNRI criteria using public stakeholders: an exercise using Amazon Mechanical Turk. J Glob Health 2019In press 10.7189/jogh.09.010702 PMC6445564 30992986

[8] Tomlinson M, Rudan I, Saxena S, Swartz L, Tsai AC, Patel V (2009). Setting priorities for global mental health research.. Bull World Health Organ.

[9] Tomlinson M, Chopra M, Sanders D, Bradshaw D, Hendricks M, Greenfield D (2007). Setting priorities in child health research investments for South Africa.. PLoS Med.

[10] Kapiriri L, Tomlinson M, Chopra M, El Arifeen S, Black RE, Rudan I (2007). Setting priorities in global child health research investments: addressing values of stakeholders.. Croat Med J.

[11] Sekar N, Shah NK, Abbas SS, Kakkar M (2011). RCZ RCZI. Research options for controlling zoonotic disease in India, 2010-2015.. PLoS One.

[12] Kapiriri L, Norheim OF (2004). Criteria for priority-setting in health care in Uganda: exploration of stakeholders’ values.. Bull World Health Organ.

[13] Shapiro DN, Chandler J, Mueller PA (2013). Using Mechanical Turk to study clinical populations.. Clin Psychol Sci.

