# Supplementary material for: Exploring individual and demographic characteristics and their relation to CHNRI Criteria from an international public stakeholder group: an analysis using random intercept and logistic regression modelling
Source: J Glob Health. 2019 Feb 15;9(1):010701. doi: 10.7189/jogh.09.010701 (PMC6377795; doi:10.7189/jogh.09.010701)
Supplement: Online Supplementary Document [file jogh-09-010701-s001.pdf]

Wazny.et al. J Glob Health 2019; 9: 010701

Online Supplementary Document

Appendix S1*. Full survey, including instructions, demographic questions, CHNRI criteria and corresponding questions and questions intended to identify malicious Turkers.*

Thank you for participating in our survey on values for research prioritisation. We are collecting information on demographics (your age, where you live, etc.) and then on what you value most, so we will be able to apply your values to influence how funding agencies choose what to give money to when we set priorities for health research around the world. Your responses will be used to influence how money is spent on research in the future and could have great impact, so thank you! Participation is completely voluntary and we will not be able to identify you in any way, as is Amazon Mechanical Turk's policy. If you have any questions about the project, please contact kerri.wazny@ed.ac.uk.

Individual and Demographic Questions

Question	Variable(s)
Age	What is your age?
Gender	What is your gender?<br>Options: Male, Female, Non-binary, Other
Country of residence	In what country do you live?
Country of birth	In what country were you born?
Urban v. Rural	Do you live in a large city or a town?<br>Options: Large city (more than 50,000 people live in your city); town (less than 50,000 people live in your town)
Ethnicity	What race/ethnicity best describes you? (Please choose only one)<br>Options: White, Black African, Black Caribbean, Other Black, Middle Eastern, Southeast Asian, East Asian, South Asian, Central/South American, Multiple Ethnicity
Marital Status	Which of the following best describes your marital status?<br>Options: Married, Widowed, Divorced, Separated, Common-law or domestic partnership/civil union, Single
Religion	What is your religion?<br>Options: Christian/Protestant/Methodist/Lutheran/Baptist, Catholic, Mormon, Greek or Russian Orthodox, Muslim, Hindu, Sikh, Buddhist, Judaism, Non-religious, Other
Employment status	What is your employment status?<br>Options: Self-employed, Employed part-time, Employed full-time, Student, Retired, Unemployed, Disabled (not able to work)
Health stakeholder	Do you work or have you worked in the health sector? (For example, as a doctor, nurse, in an organization that works in health through donations or research, as a health economist, dietician, sports therapist, etc.)
Educational attainment	What is your highest level of education?<br>Options: No formal education, Primary school, Some Secondary school, Secondary school, Some College, College (2 year degree), University (4 year degree), Graduate level

	degree (Masters or equivalent), Professional degree (i.e. J.D., M.D.), Postgraduate degree (PhD)
Political affiliation	When it comes to politics, would you describe yourself as liberal, conservative, or neither liberal nor conservative?<br>Options: Extremely liberal, moderately liberal, slightly liberal, neither liberal nor conservative, slightly conservative, moderately conservative, extremely conservative
Overcrowding	How many people currently live in your household?<br>(enter a number)
Household earnings	What is the total amount of *yearly* earnings for your *household*? (please enter a whole number in the currency of your country of residence)
Self-reported health status	How would you describe your health on a scale of 1-5, with 1 being very poor and 5 being excellent

CHNRI Criteria:

Instructions: Please use the scale of 1-5 to rank the following questions. The questions relate to research in health services, not just in your country, but throughout the world. Keep in mind that the values these questions refer to could include if you were participating in a study, what would be factors that would motivate you to participate or, generally, what kind of research you would think is important to do.

**Criterion**	**Question**
Equity	How important is it for the research to help health access become more fair between people?
Disease burden reduction	How important is it for the research to result in less disease? For example, if researchers were studying heart disease, could they reduce people having heart attacks?
Answerability	How important is it for the researchers to be able to create a study to properly answer their research question?
Effectiveness	How important is it that the results of the research have an impact and will people (including doctors, nurses, and patients) actually use them?
Deliverability	How important is it that the results of the research are affordable to those who need them and to those who pay for the results (for example, the national or local government, or patients)?
Feasibility	How important is it for the researchers to have enough time, funding and skilled staff to carry out the research?
Likelihood to fill a knowledge gap	How important is it for this research to result in new information?
Cost	How important is it for the results of this research to be less expensive than similar alternatives currently available? For example, if the research is looking at a drug for blood pressure, will the new drug be less expensive than the ones available now?
Sustainability	How important is it for the results to be long-lasting?
Acceptability/Issues surrounding use	How important is it for the research and the results of the research to be respectful to local beliefs and cultural practices?
Scale	How important is it that the results of the research will be widely available (for example, the results will be available throughout the country)?

Likelihood to attract national policy attention/Translational value	How important is it that the results of this research eventually turn into policy? For example, if a research is looking into a better way to identify diabetes, the government adopts the results and uses them to find people who have diabetes.
Implementation	How important is it that the intervention or results of this research can be changed to fit different groups of people (for example, different countries, regions in countries, or religions)? For example, medications that have cow-based products cannot be used in Hindu populations because of religious reasons – is it important for medicines not to use cow-based products?
Technical possibility	How important is it that if the research involves technology, that the technology is easy to use and not expensive to develop?
Innovation	How important is it that the research is trying to make something better than what is currently being used?

Malicious Worker Questions

These questions will be scattered throughout the survey. The aim is to identify malicious workers and to exclude their answers from the analysis.

Please select number 3	Multiple choice or drop-down:<br>Options: 33, 4, 5, 3, 63
What is 1 + 1	Multiple choice or drop down:<br>Options: 1, 5, 11, 2, 8
What is this survey for?	Multiple choice or drop down:<br>Options: animals, health, technology, environment
Please select the star for ‘very important’	Star-scale (Likert scale)
Please select the star for ‘slightly important’	Star-scale (Likert scale)
Please select the fourth star	Star-scale (Likert scale)
